# Recurrent Seizures in an Adolescent Female With Extensive Intracranial Calcifications: A Case Report of Fahr’s Syndrome Secondary to Hypoparathyroidism

**DOI:** 10.7759/cureus.78780

**Published:** 2025-02-09

**Authors:** Yitayew E Mohammed, Kaleab H Gebreselassie, Elfazin H Sesega, Ayenew A Wolie, Bargicho J Ahmed

**Affiliations:** 1 Internal Medicine, Worabe Comprehensive Specialized Hospital, Worabe, ETH; 2 Surgery, Worabe Comprehensive Specialized Hospital, Worabe, ETH; 3 Internal Medicine, Hawassa University College of Medicine and Health Sciences, Hawassa, ETH; 4 Emergency Medicine, Worabe Comprehensive Specialized Hospital, Worabe, ETH; 5 Radiology, Worabe Comprehensive Specialized Hospital, Worabe, ETH

**Keywords:** basal ganglia, cataract, fahr’s syndrome, hypocalcemia, hypoparathyroidism, intracranial calcification, seizure

## Abstract

Fahr’s syndrome is a rare, progressive, neuropsychiatric disorder characterized by bilateral and symmetrical calcifications over the basal ganglia and other parts of the brain, leading to a wide range of clinical manifestations ranging from neurologic symptoms of movement disorders, seizures, and cerebellar dysfunction to neuropsychiatric symptoms such as dementia, psychosis, and mood disorders. The widespread calcific deposits within the brain tissue that characterize Fahr’s syndrome develop secondary to different underlying conditions such as parathyroid disorders, brain infections, and toxic exposures. Hypoparathyroidism, a rare disorder of calcium and phosphate metabolism, is the most commonly identified etiology of Fahr’s syndrome.

In this case, we report a case of a 17-year-old female patient who presented with status epilepticus. Her past medical history was positive for intermittent episodes of generalized tonic-clonic seizures for the past year. Upon presentation, she had a decreased level of consciousness with a Glasgow Coma Scale score of 9 (eye-opening = 2, verbal response = 2, motor response = 5). Chvostek’s and Trousseau’s signs were positive. Initial laboratory workup revealed severe hypocalcemia, hyperphosphatemia, and markedly low parathyroid hormone levels. Computed tomography of the brain showed extensive, bilateral, symmetrical calcifications over the basal ganglia, thalami, corona radiata, and dentate nuclei. She was subsequently diagnosed with Fahr’s syndrome secondary to hypoparathyroidism and was managed with calcium gluconate, vitamin D, and sodium valproate, which improved her condition. A slit lamp examination of the eyes revealed a bilateral posterior subcapsular cataract more severe in the right eye, for which small incision cataract surgery was performed on her right eye.

This case report underscores the importance of considering a diagnosis of Fahr's syndrome in adolescent patients with a history of seizures and unexplained intracranial calcifications on brain imaging. It also emphasizes the necessity of thorough clinical assessment and laboratory tests to identify the underlying cause, as the treatment of Fahr’s syndrome primarily focuses on identifying and managing the underlying etiology.

## Introduction

Fahr’s syndrome is a rare, progressive, neuropsychiatric disorder characterized by bilateral and symmetrical calcifications in different parts of the brain, particularly the basal ganglia. The abnormal calcific deposits may also appear in the thalamus, subcortical white matter, cerebral cortex, cerebellum, and dentate nucleus [1,2]. These calcifications can result in a wide range of clinical manifestations, including neurologic symptoms such as basal ganglia movement disorders, seizures, cerebellar dysfunction, and speech difficulties, as well as neuropsychiatric symptoms like dementia, schizophrenia-like psychosis, and mood disorders [1-3].

Fahr’s syndrome should be distinguished from Fahr’s disease, a different clinical entity with several similarities. Both conditions are characterized by pathologic bilateral basal ganglia calcifications that result in neuropsychiatric sequelae; however, the two conditions have distinct and critical differences in etiology, lesion location, prognosis, and treatment [4]. Fahr’s disease refers to primary or idiopathic forms of intracranial calcifications that occur without any known underlying conditions, while the calcifications in Fahr’s syndrome arise due to various secondary conditions [1,2,4]. Diseases of the parathyroid gland, particularly hypoparathyroidism, are the most commonly identified etiologic conditions associated with Fahr’s syndrome [2,5].

Fahr's syndrome is a rare condition with a limited number of cases reported in the literature, and its pathogenesis, etiology, and treatment are not yet well characterized. Moreover, the condition is even more rare in adolescents. Herein, we report a case of Fahr’s syndrome secondary to hypoparathyroidism in an adolescent female who presented with status epilepticus. This case report aims to be a valuable addition to the efforts of the global medical community for a better understanding of the etiology, pathophysiology, clinical presentation, and diagnosis of this rare condition.

## Case presentation

A 17-year-old female patient presented to the emergency department with frequent episodes of generalized tonic-clonic seizures lasting 14 hours, without regaining consciousness between episodes. The patient also reported experiencing a globalized headache of four days' duration, associated with fatigue and low-grade fever, but with no history of vomiting, limb weakness, or loss of sensation.

Over the past year, the patient had a history of intermittent generalized tonic-clonic seizures occurring at least once per month, which had become more frequent over the past month. Two weeks before her current admission, she visited a local clinic for the recent worsening of the seizure episodes, and she was started on oral phenobarbital 100 mg once daily.

The patient is a high school student and had no history of cognitive decline, decrement in concentration, recent change in behavior, anxiety, or features of depression. She had no history of neck surgery, neck radiation, head trauma, smoking, alcohol or substance use. She also denied any family history of seizures or hypoparathyroidism.

Upon presentation to the emergency department, the patient’s vital sign recordings were within the normal range. On neurological examination, she had a decreased level of consciousness with a Glasgow Coma Scale score of 9 (eye-opening = 2, verbal response = 2, motor response = 5). Her pupils were mid-sized and equally reactive to light. There were no signs of cranial nerve abnormalities or focal neurological deficits. Deep tendon reflexes were 2/4 bilaterally at the patella and ankle. The plantar reflex was down-going bilaterally. Signs of meningeal irritation were negative. Chvostek’s and Trousseau’s signs were positive. A slit-lamp examination of the eyes revealed a bilateral posterior subcapsular cataract, which was more severe in the right eye.

The patient’s initial serum electrolyte analysis revealed a total calcium concentration of 0.44 mmol/L (reference range, 1.9-2.75 mmol/L), phosphorus concentration of 1.65 mmol/L (reference range, 0.81-1.45 mmol/L), and magnesium concentration of 0.54 mmol/L (reference range, 0.66-1.07 mmol/L). Serum parathyroid (PTH) concentration was <4 pg/mL (reference range, 15-68.3 pg/mL), and serum 25-hydroxy vitamin D concentration was 12.8 ng/mL (reference range, 20-100 ng/mL). Her infectious workup was negative for any organisms. A summary of her initial and follow-up laboratory investigations is shown in Table 1.

**Table 1:** Summary of the results of the laboratory investigations. WBC, white blood cells; AST, aspartate aminotransferase; ALT, alanine aminotransferase; ALP, alkaline phosphatase; PTH, parathyroid hormone; 25-OH vitamin D, 25-hydroxyvitamin D; T3, triiodothyronine; T4, tetraiodothyronine; TSH, thyroid-stimulating hormone; FSH, follicle-stimulating hormone; HbA1c, hemoglobin A1c; CSF, cerebrospinal fluid; HIV, human immunodeficiency virus; VDRL, venereal disease research laboratory; ANA, antinuclear antibody; NA, not available

Laboratory investigation (unit)	On admission	One month after admission	Three months after admission	Reference range
WBC (per μL)	5,200	9,280	5,770	2,900-20,000
Hemoglobin (gm/dL)	12.9	9.5	12.8	12-16.5
Platelet count (per μL)	84,000	82,000	285,000	150,000-450,000
ALT (IU/L)	19.2	18	14.5	0-41
AST (IU/L)	72.2	58	22.5	0-40
ALP (IU/L)	NA	88	NA	40-130
Serum albumin (mg/dL)	2.18	2.54	3.69	3.5-5.2
Urea (mg/dL)	21.5	31.8	NA	16.6-48.5
Creatinine (mg/dL)	0.55	1.11	0.46	0.0-1.1
Sodium (mmol/L)	137	143	NA	136-145
Potassium (mmol/L)	4.42	3.59	NA	3.5-5.1
Chlorine (mmol/L)	97.4	102.7	NA	98-107
Total calcium (mmol/L)	0.44	1.19	2.21	1.9-2.75
Phosphorous (mmol/L)	1.65	1.43	NA	0.81-1.45
Magnesium (mmol/L)	0.54	0.81	NA	0.66-1.07
PTH (pg/mL)	<4			15-68.3
25-OH, vitamin D (ng/mL)	12.8			20-100
T3 (ng/mL)	0.942			0.846-2.02
T4 (μg/dL)	6.05			5.13-14.06
TSH (mIU/L)	1.98			0.27-4.20
FSH (IU/L)	4.86			Follicular (3.5-12.5)
Prolactin (ng/mL)	6.12			4.06-23.3
HbA1c (%)	4%			<5.7%
CSF analysis	Unremarkable			-
Hepatitis B surface antigen	Negative			Negative
Hepatitis C antibody	Negative			Negative
HIV test	Negative			Negative
VDRL	Negative			Negative
ANA	Negative			Negative

**Figure 1:**
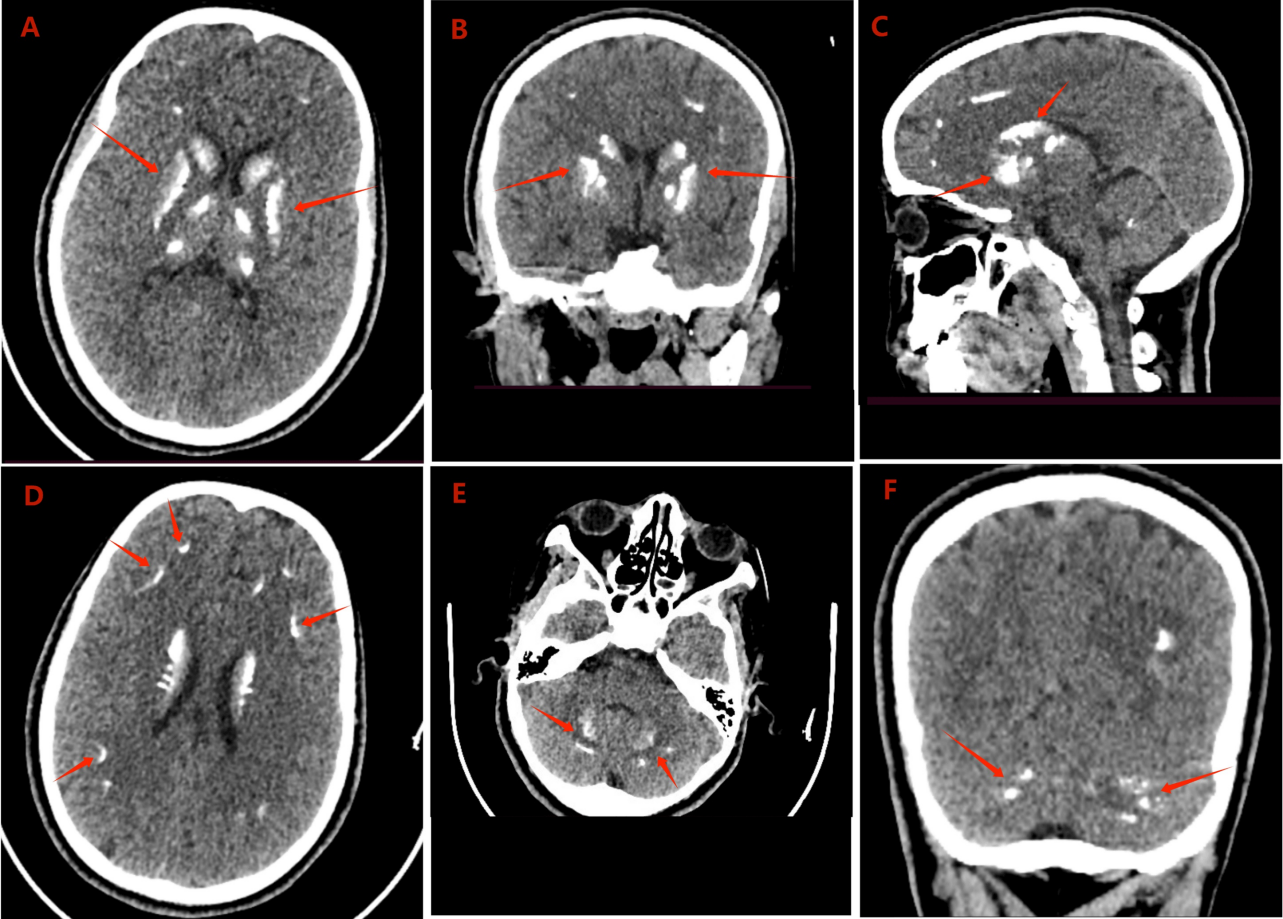
A non-contrast brain CT scan showed bilateral, symmetrical calcifications in the basal ganglia, thalami, corona radiata, and dentate nuclei. (A) Axial, (B) coronal, and (C) sagittal images showing bilateral calcifications of the basal ganglia and thalami (red arrows). (D) Axial image showing bilateral calcifications of the corona radiata (red arrows). (E) Axial and (F) coronal images showing bilateral calcifications of the dentate nuclei (red arrows).

**Figure 2:**
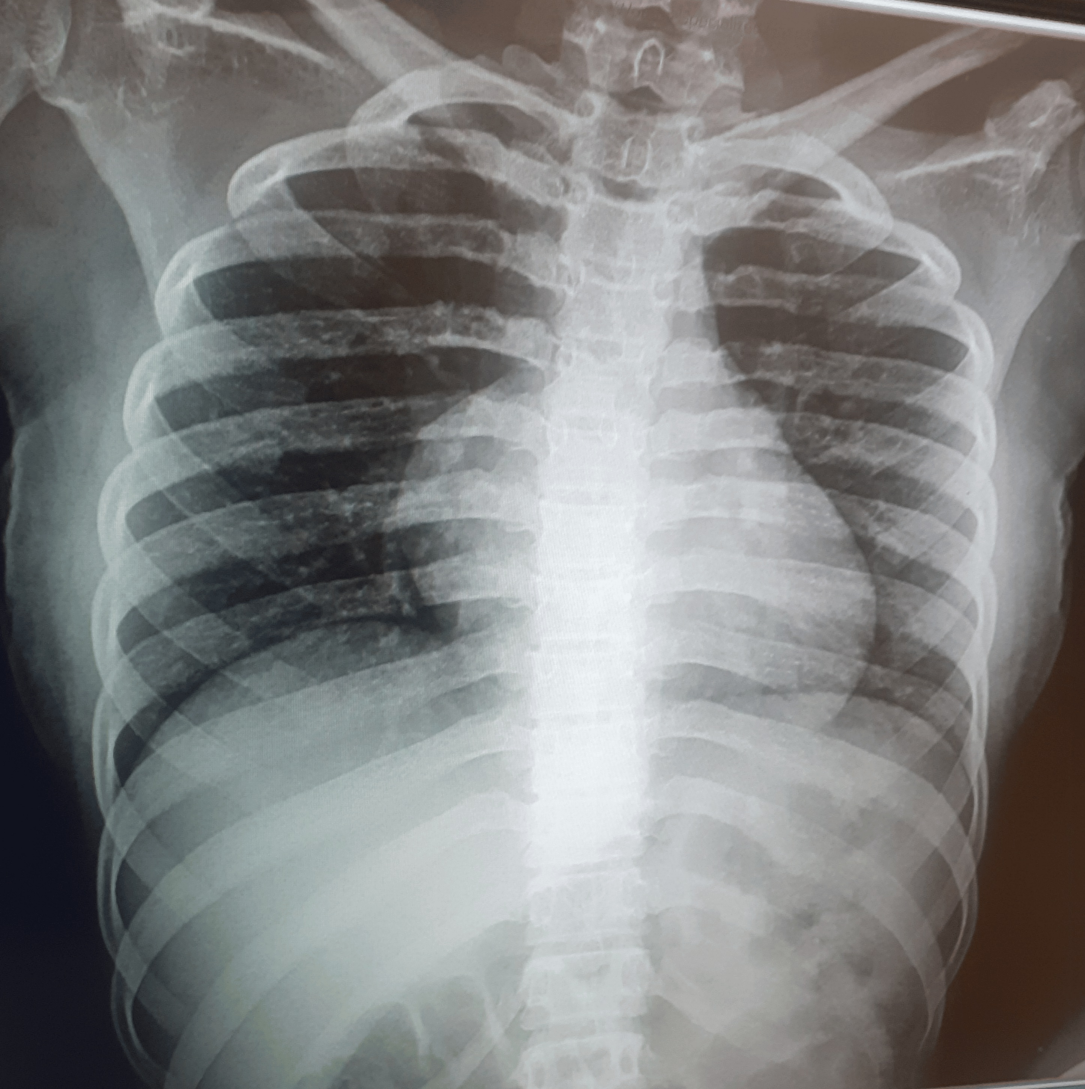
Chest X-ray showing normal findings.

**Figure 3:**
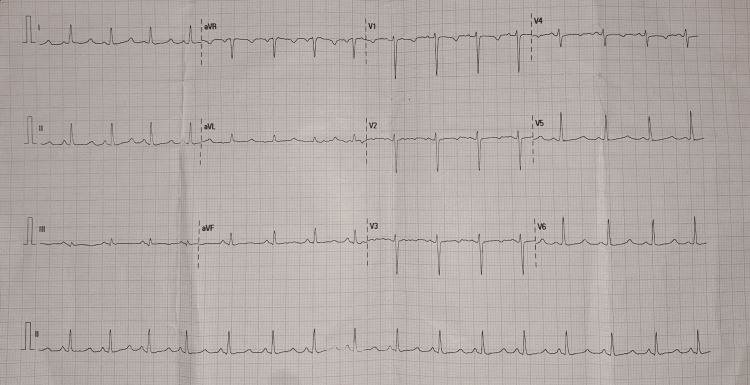
Electrocardiogram (EKG) recording showing normal tracing with a normal QT interval.

A non-contrast computed tomography (CT) scan of the brain revealed bilateral, symmetrical, large areas of dense calcification over the basal ganglia, thalami, corona radiata, and dentate nuclei (Figures 1A-1F). Chest X-ray (Figure 2), abdominal ultrasound, thyroid ultrasound, and echocardiography findings were unremarkable. Electrocardiogram (EKG) recording showed normal tracing with a normal QT interval (Figure 3).

Based on the patient’s clinical presentation, brain imaging results of bilateral and symmetric intracranial calcifications, and laboratory results showing severe hypocalcemia, hyperphosphatemia, and a low PTH level, a diagnosis of Fahr’s syndrome secondary to hypoparathyroidism was made. A thorough clinical history, physical examination, and laboratory investigations to determine the cause of hypoparathyroidism revealed no history of thyroid surgery or neck radiation therapy, no anomalies suggestive of genetic forms of hypoparathyroidism (such as dysmorphic facies, short stature, or developmental delay), and no findings indicative of autoimmune hypoparathyroidism (such as mucocutaneous candidiasis, adrenal insufficiency, hypothyroidism, diabetes mellitus, or positive autoimmune markers). Although genetic disorders can rarely cause genetic forms of isolated hypoparathyroidism [6], genetic testing to exclude such etiologies was not sent to our patient due to resource limitations.

The patient was initially managed with intravenous calcium gluconate to control the seizure and rapidly correct the severe symptomatic hypocalcemia. After initial improvement of the serum calcium level and resolution of the hypocalcemic signs, she was started on oral calcium carbonate. Parenteral magnesium sulfate was administered to manage the coexisting hypomagnesemia. Additionally, she was started on oral sodium valproate, 200 mg twice daily.

On the second day of admission, the patient developed an extensive erythematous rash that rapidly progressed to form flaccid blisters and erosions over her face, chest, back, limbs, oral mucosa, and nasopharynx. Simultaneously, her serum creatinine level rose to 3.99 mg/dL. She was diagnosed with toxic epidermal necrolysis complicated by acute kidney injury (AKI). Phenobarbital, which she had been taking for two weeks before admission, was identified as the culprit. With supportive care, including fluid replacement and wound care, her skin condition and AKI gradually improved during her hospital stay.

The patient was discharged from the hospital after a 50-day stay with oral calcium carbonate 1,000 mg three times daily, oral vitamin D-3 (cholecalciferol) 50,000 IU weekly, and oral sodium valproate 200 mg twice daily. Her follow-up after three months revealed no history of seizure episodes and normalization of serum calcium level. Two months after discharge, a small incision cataract surgery was done on her right eye to extract the severe posterior subcapsular cataract.

## Discussion

Fahr’s syndrome is a rare neuropsychiatric disorder with a prevalence of <1 in 1,000,000 and commonly affects individuals in their third and fourth decades of life [1]. It is characterized by bilateral and symmetrical calcifications in different areas of the brain, particularly the basal ganglia. The widespread calcific deposits within the brain tissue could result in a spectrum of neurologic and neuropsychiatric manifestations [1,2].

A diagnosis of Fahr’s syndrome should be considered in patients with: (1) signs and symptoms of basal ganglia movement disorder, cognitive impairment, gait disorder, cerebellar abnormality, speech dysfunction, or psychiatric disorder; (2) brain imaging finding of symmetrical, bilateral intracranial calcification particularly over the basal ganglia; and (3) presence of an underlying condition associated to the development of the calcific deposits [4]. Although several conditions have been implicated in causing Fahr’s syndrome, the most commonly identified etiologic conditions are parathyroid gland disorders (hypoparathyroidism, pseudohypoparathyroidism), brain infections (brucellosis, toxoplasmosis, HIV), and toxic exposures (lead, carbon monoxide) [1,2,4,7].

Non-contrast CT of the brain is the imaging of choice in patients with Fahr's syndrome to localize and assess the extent of the intracranial calcifications [1]. The clinical diagnosis of Fahr’s syndrome is often challenging due to the variability and non-specificity of neurologic manifestations, as well as the lack of a clear anatomoclinical correlation between the location and severity of calcifications and the degree of neurologic impairment [5,8]. Moreover, the heterogeneity of the neuropsychiatric and neuroradiological symptoms attributable to the underlying etiology could delay the clinical suspicion of Fahr’s syndrome. Therefore, a thorough assessment of the patient’s clinical history, physical findings, laboratory results, and imaging findings is essential for a timely diagnosis of Fahr’s syndrome and its etiology [2].

Hypoparathyroidism, a rare metabolic disorder characterized by hypocalcemia, hyperphosphatemia, and low PTH level [6], is the most commonly identified etiologic condition associated with the development of Fahr’s syndrome [2,5]. Prolonged duration of low serum calcium/phosphorus ratio, poor calcium control, and hyperphosphatemia in patients with chronic hypoparathyroidism are believed to be major contributing factors for the development and progression of intracranial calcifications. Management of this metabolic imbalance by maintaining a higher calcium/phosphorus ratio has been shown to reduce the risk of development and progression of the calcifications, highlighting the role of early diagnosis and adequate therapy of hypoparathyroidism to prevent this neuropsychiatric disorder [9].

Hypoparathyroidism is most commonly caused by the destruction of the parathyroid glands due to neck surgery or autoimmune conditions. Genetic disorders could rarely cause hypoparathyroidism, which may occur as part of syndromic disorders or as a non-syndromic solitary endocrinopathy (isolated or idiopathic hypoparathyroidism) [6,10]. According to a review of 223 cases with Fahr’s syndrome due to hypoparathyroidism, idiopathic/familial hypoparathyroidism was the most common etiology of Fahr’s syndrome followed by post­surgical hypoparathyroidism [5]. In another study by Goswami et al., the prevalence of basal ganglia calciﬁcation in patients with idiopathic hypoparathyroidism was 73·8% [9].

The diagnosis of Fahr’s syndrome due to hypoparathyroidism is usually delayed, compared to Fahr’s syndrome due to other causes. The average delay between symptom onset and diagnosis is approximately a decade [5]. The most common symptoms at onset in patients with Fahr’s syndrome due to hypoparathyroidism are seizure and tetany, followed by movement disorders and neuropsychiatric symptoms. The symptoms may be caused by the abnormal neuronal excitation of the basal ganglia due to hypocalcemia, structural changes of the basal ganglia and their connections due to calcifications, or a combination of both [1,5,9,11].

In patients with chronic hypoparathyroidism, other complications besides intracranial calcifications such as renal function impairment, nephrocalcinosis, nephrolithiasis, cardiovascular diseases (QT prolongation, cardiomyopathy), and cataracts should also be monitored and managed [6]. In our patient, a slit-lamp examination of the eyes revealed bilateral posterior subcapsular cataracts. Hypoparathyroidism has been shown to increase the risk of posterior subcapsular cataracts likely due to elevated calcium × phosphorus product occurring within the lenses of the eyes [6]. In a study by Goswami et al. on 145 patients with idiopathic hypoparathyroidism, the prevalence of cataracts was 51% [9].

To date, there have been no specific treatment guidelines for the management of Fahr’s syndrome. Treatment primarily focuses on symptomatic management and addressing the underlying cause [4]. The mainstay of treatment for patients with hypoparathyroidism is the correction of hypocalcemia with calcium and vitamin D preparations, which often leads to significant improvement in neurologic symptoms [11,12]. Long-term management of hypoparathyroidism should aim to maintain serum calcium levels within the low-normal range (8.0-9.0 mg/dL) and serum phosphorus levels within the high-normal range and prevent significant hypo- or hypercalcemia. In patients with low or absent PTH, calcium absorption is dependent on daily calcium and active vitamin D intake. Therefore, patients with hypoparathyroidism require a consistent daily intake of calcium and active vitamin D to maintain target serum calcium levels [6].

## Conclusions

In summary, although Fahr’s syndrome is a rare condition commonly affecting middle-aged and older individuals, our case report highlights the importance of considering a diagnosis of Fahr’s syndrome in younger patients presenting with neuropsychiatric symptoms. The underlying cause of Fahr’s syndrome in our 17-year-old female patient was hypoparathyroidism. The successful management of the patient’s condition with the prompt correction of her severe hypocalcemia underscores the vital role of early identification and management of the underlying cause in the overall care of patients with Fahr’s syndrome. Furthermore, this case emphasizes the necessity of meticulous assessment of patients with hypoparathyroidism for the early detection and management of other complications, such as cataracts.
